# A Switchable Deep Eutectic System Based on Diarylethene

**DOI:** 10.1021/acsomega.5c04216

**Published:** 2025-10-21

**Authors:** Hugo Cruz, Noémi Jordão, Sara Santiago, Silvia Mena, Andreia F. M. Santos, M. Teresa Viciosa, Karolina Zalewska, Luis C. Branco, Jordi Hernando, Gonzalo Guirado

**Affiliations:** † LAQV-REQUIMTE, Department of Chemistry, NOVA School of Science and Technology, 426467 NOVA University of Lisbon, Caparica 2829-516, Portugal; ‡ INL − International Iberian Nanotechnology Laboratory, Av. Mestre José Veiga, Braga 4715-330, Portugal; § Departament de Química, 16719 Universitat Autònoma de Barcelona, Cerdanyola Del Vallès, Barcelona 08193, Spain; ∥ Centro de Química Estrutural, Institute of Molecular Sciences, Instituto Superior Técnico, University of Lisbon, Av. Rovisco Pais, Lisbon 1049-001, Portugal

## Abstract

Deep eutectic solvents (DESs) have emerged as promising alternative solvents for a wide range of applications, such as extraction and separation processes. To broaden their functionality, the development of responsive DESs (RDESs) has attracted particular interest due to their ability to abruptly change their properties in response to external stimuli. Herein, we aim to prepare a new RDES comprising a photo- and electrochromic dicarboxylic diarylethene derivative (DTE) and quadrol (Q). The prepared system exhibits a color change from yellowish to pinkish-red under UV irradiation due to the photoisomerization of DTE, which can be reverted by visible light or oxidative electrolysis. Interestingly, this process also alters the strength of the hydrogen bonds between DTE and Q, leading to an externally controlled change in the thermal and electrical properties of the eutectic mixture. Therefore, these results demonstrate the capacity to photo- and electromodulate the behavior of DESs by incorporating stimuli-responsive units, thereby opening new perspectives toward the design of solvents with tunable properties for a variety of industrial and materials science applications.

## Graphical abstract

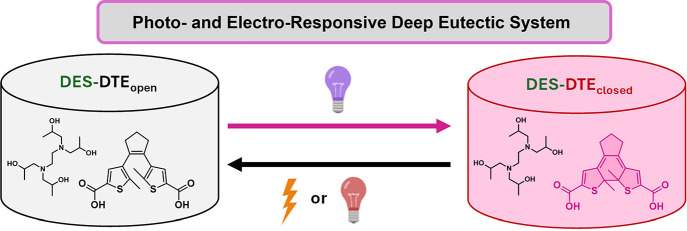

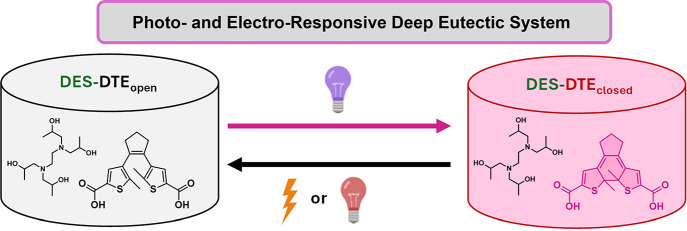

## Introduction

Currently, our society recognizes the need for more sustainable, eco-efficient, and eco-friendly chemical and engineering processes due to the intensive use of natural resources.^1^ Considering that most chemical processes require the usage of conventional organic solvents, which can negatively impact pollution, energy consumption, air quality, and climate change, it is necessary to develop more sustainable solvents.^2^ In this context, Pollet et al. proposed that “solvents must address simultaneously reaction efficiency, product separation and recyclability” to be considered competitive and environmentally conscious, contributing to the optimization of the overall process as well as its cost-effectiveness.^3,4^

In the past decades, different types of nonconventional solvents have been developed to meet these requirements. This is the case with switchable solvents (SSs), i.e., solvents that reversibly change their properties in response to an external stimulus (e.g., CO_2_, pH, temperature), which have enabled improved reaction conditions as well as product separation for some selected processes.^3–5^ For instance, one of the main applications of SSs is the development of CO_2_-responsive systems, where CO_2_ acts as a trigger to induce changes in the solvent properties, such as hydrophobicity and polarity, which can be exploited for a variety of applications.^6^ More recently, another class of disruptive solvents has emerged that is composed of eutectic systems (ESs), as is the case for deep eutectic solvents (DESs). Usually, ESs are obtained by the complexation of a hydrogen-bond acceptor (HBA) and a hydrogen-bond donor (HBD), without additional purification steps.^7^ These systems are an alternative to ionic liquids for the preparation of electrolytic media without the need for conventional organic solvents and supporting electrolytes, as they can combine rather high electrical conductivity with several additional advantages, such as easier preparation, eco-friendliness, and low-cost starting materials.^7,8–10^ As a result, ESs and DESs are being explored in a wide range of applications, particularly in materials science, including electroplating and electrodeposition processes, electrocatalysis, supercapacitors and batteries, electrolytes for electrochromic devices, photochromic nanocomposites, and bioinspired and responsive materials.^7,11–32^

To further expand the functionality of SSs and DESs, the development of new solvents that combine the best of both worlds has been proposed in recent years, giving rise to the so-called responsive DESs (RDESs), whose properties can be modulated with external stimuli.^33^ This is the case for intrinsically electroresponsive DESs based on viologen derivatives, which behave as multifunctional materials that can act as both electrolytes and electrochromogens in electrochromic devices.^12^

Another very relevant stimulus for the preparation of responsive systems is light, which is used for manipulating the behavior of several photochromic materials currently exploited in ophthalmics (e.g., commercially available light-responsive lenses for sunglasses), photo-optical switching devices (e.g., optical memories, sensors, color switches, encryption/decryption devices, and actuators or molecular machines), light filters, as well as in textile, dyes, and cosmetic industries.^34,35^ All these materials undergo a color change upon light excitation due to the reversible photoinduced transformation between two different states, which not only modifies their absorption spectra but also other physical and chemical properties.^36^ Among the different light-responsive molecular systems designed to achieve this behavior, diarylethenes (DAEs) stand out, as they exhibit reversible photochemical reactivity, even in the solid state, with high efficiency, fatigue resistance, and low thermal reversibility, which can be tuned by modifying the functional groups introduced.^37,38^ In particular, DAEs undergo a 6π-electron photocyclization reaction from their initial open isomer, generally colorless, to the colored closed state that is promoted by irradiation with UV light, i.e., ring-closure process. Then, back-photoisomerization takes place under visible light irradiation through a photocycloreversion reaction—i.e., ring-opening process.^38,39^ Besides light, the cyclization and/or cycloreversion reactions of DAEs can also be promoted by electrochemical oxidation or reduction processes, a redox-switchable behavior that is highly dependent on the substituents present on the DAE moiety.^40–48^

Inspired by these attractive features of diarylethenes, in this work, we report their use for the preparation of a novel stimuli-responsive ES (ES-1), whose chemical, thermal, and electrical properties can be modulated with light and redox potentials. To achieve this goal, two different components were mixed in ES-1: *N*,*N*,*N*,′*N*′-tetrakis (2-hydroxypropyl)­ethylenediamine, known as quadrol (Q), as a hydrogen bond acceptor; and a photoelectrochromic DAE-based dicarboxylic acid (DTE) as a hydrogen bond donor, which contains a switchable dithienylethene core (Figure 1). Interestingly, DTE is a Brønsted acid whose acidity/ability to form hydrogen bonds can be tuned with light and electrons due to the change in electronic communication that occurs between the two external carboxylic acid groups upon photoisomerization: they are insulated from each other in the open state and become selectively conjugated in the closed isomer, which has been reported to cause an increase in acidity after photocyclization.^49,50^ As a result, switching between the open and closed isomers of DTE should reversibly modulate its hydrogen bonding interactions with Q, thus modifying the physicochemical properties of ES-1. It is worth mentioning that the ability of these responsive deep eutectic solvents (RDESs) to reversibly change color, polarity, and hydrogen bonding strength under photochemical or electrochemical stimuli makes them ideal candidates for the selective and tunable extraction of target compounds, for their use as active electrolytes in electrochromic devices, or for the on-demand modulation of catalytic activity and selectivity—paving the way for more efficient and controllable chemical processes.

**Figure 1:**
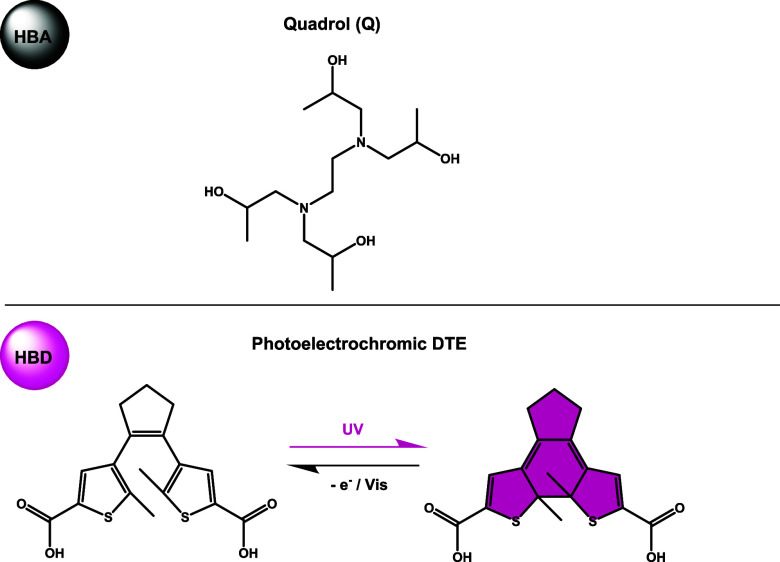
Chemical structures of HBA and HBD selected to form ES-1.

## Experimental Section

### Chemicals

*N*,*N*,*N*,′*N*′-Tetrakis (2-hydroxypropyl)­ethylenediamine (Q, > 98%), methanol (MeOH), and acetonitrile (ACN, puriss. electrochemical grade) were acquired from Sigma-Aldrich and used without further purification. Tetrabutylammonium hexafluorophosphate ([TBA]­PF_6_ puriss. electrochemical grade) was purchased from Fluka and dried overnight at 80 °C before use. The photoelectrochromic diacid 1,2-bis­(5′-carboxy-2’-methylthien-3′-yl)-cyclopentene (DTE) was synthesized according to the procedures previously reported by us.^48,51^

### Preparation of the Eutectic System, ES-1

To prepare ES-1, 1 equiv of DTE and 12 equiv of Q were stirred at 60 °C for 8 h until a yellowish viscous liquid was obtained. ^1^H NMR (400.13 MHz, MeOD, 25 °C): δ = 7.38 (s, 2H) 3.37–3.33 (m, 48H), 3.00–2.29 (m, 144H), 1.82 (s, 6H), 1.14–1.11 (m, 144H) ppm.

### Characterization of the Eutectic System, ES-1

#### ^1^H Nuclear Magnetic Resonance (^1^H NMR)

^1^H NMR spectra were recorded on a Bruker AMX400 spectrometer using MeOD as a deuterated solvent, and the chemical shifts were reported upfield in parts per million.

#### Differential Scanning Calorimetry (DSC)

Thermal studies were carried out using a DSC Q2000 from TA Instruments Inc. (Tzero DSC technology) coupled to an RCS 90 cooling system and operating in the Heat Flow T4P option. Measurements were performed under anhydrous conditions, purged with 50 mL min^–1^ of high-purity nitrogen flow. DSC Tzero apparatus calibration was done in the temperature range between −90 and 200 °C. Enthalpy (cell constant) and temperature calibration were based on the melting peak of the indium standard (*T*_m_ = 156.60 °C) supplied by TA Instruments (lot number: E10W029). For each experiment, the samples were first dried under vacuum. Then, ∼4–7 mg were weighed and encapsulated in a hermetic Tzero aluminum pan and lid. All capsules were sealed before the analysis, and the respective lids were perforated to prevent pressure build-up due to water evaporation. The experimental procedure consisted of first equilibrating all samples at 20 °C before subjecting them to several cooling and heating runs between −90 and 120 °C. Three scans at 10 °C min^–1^ were conducted to remove adsorbed water, record the phase transformations, and confirm the results obtained in the second run. Moreover, cooling/heating cycles at 5 °C min^–1^ and 20 °C min^–1^ were also performed. Data treatment was carried out through Universal Analysis 2000 software by TA Instruments Inc.

#### Dielectric Spectroscopy (DS)

The electrical conductivity of Q and ES-1_open/closed_ was studied by Dielectric Spectroscopy (DS). The samples were placed between two stainless steel electrodes (10 mm diameter) in a BDS 1200 parallel plate capacitor, with two 50 μm silica spacers used to maintain the sample thickness. The sample cell was mounted on a BDS 1100 cryostat. Temperature control was ensured by a Quatro Cryosystem controller and maintained to be within ±0.5 °C (all modules supplied by Novocontrol). Measurements were carried out using an Alpha-N analyzer from Novocontrol, covering a frequency range from 10^–1^ to 10^6^ Hz with an AC voltage of 1 [Vrms]. After a cooling ramp from 20 to −120 °C at 10 °C min^–1^, a set of isothermal spectra was collected: from −120 to −50 °C, every 5 degrees; from −48 to 80 °C, every 2 degrees; and, finally, from 85 to 120 °C, every 5 degrees (exceptionally, for quadrol, spectra were also recorded every 5 degrees between 50 and 80 °C ). This series corresponds to the “hydrated” state. Then, the samples were cooled again to −100 at 10 °C min^–1^, and a second set of isothermal spectra was acquired in the same temperature steps. This second series corresponds to the “dried” state.

#### Photo- and Electrochemical Studies

Cyclic voltammetry (CV) measurements were carried out on a CH Instrument 660E potentiostat. All electrochemical studies in solution were performed using a platinum mesh and wire as the working and counter electrodes, respectively, and a saturated calomel electrode (SCE) as a reference electrode. The selected supporting electrolyte was a solution of acetonitrile (ACN) containing tetrabutylammonium hexafluorophosphate (0.1 M [TBA]­PF_6_). Additional CV measurements were conducted on screen-printed electrodes comprising ITO, carbon, and Ag as the working, counter, and pseudoreference electrodes, respectively. Optical parameters were determined by using spectroelectrochemical techniques. A VSP100 potentiostat was coupled to an L12090 Hamamatsu spectrophotometer; both instruments were controlled and synchronized using an EC-Lab V9.51 and Biokine 32 V. 4.46 software. The spectroelectrochemical experiments were performed using an electrochemical cell with 1 mm optical path length and applying controlled potential electrolysis; hence, a constant reduction potential was first applied, followed by a constant oxidation potential. The quantity of ES-1_closed_ photogenerated from ES-1_open_ was estimated by cyclic voltammetry performed in solution through the current intensity ratio between ES-1_closed_ and ES-1_open_, which was normalized with the respective number of electrons involved in the corresponding electron transfer processes.

## Results and Discussion

### Synthesis and Structural Analysis of the Eutectic System, ES-1

ES-1 was designed to be an intrinsically photo- and electrochromic material by selecting quadrol as HBA and the well-known photoelectrochromic diarylethene 1,2-bis­(5′-carboxy-2’-methylthien-3′-yl)-cyclopentene as HBD. For ES-1 preparation, Q and DTE were mixed in a 12:1 molar ratio and stirred under heating (60 °C) for 8 h until a yellow viscous liquid was obtained. The initial feed ratio was preserved in the final material, as confirmed by ^1^H NMR spectroscopy. It is important to note that no formation of a solid salt, resulting from acid–base reaction between the amino groups of Q and the carboxylic acid moieties of the open DTE unit, was observed. Instead, a pure liquid mixture was obtained. This suggests that the carboxylic acid groups may participate in hydrogen bonding interactions with Q, probably through both its hydroxyl and amino moieties.

### Photo- and Electroinduced Responses of the Eutectic System, ES-1

To investigate whether the photoelectrochromic properties of DTE^49–51^ were reproduced in ES-1, we conducted optical, electrochemical, and electro-optical studies in both solution (5:1 v/v ACN:MeOH, Figure S1) and neat (Figure 2) conditions. In its initial state, where DTE units remained in their open form (ES-1_open_), ES-1 showed strong UV absorption and only a small absorption tail in the visible region, which provided a pale-yellow color to the neat system that was attenuated upon dilution. In contrast, irradiation with UV light (254 nm) induced a sudden coloration change to pink-red, characteristic of the closed DTE isomer, corresponding to the photocyclization of the DTE units of ES-1 (ES-1_closed_). In particular, a new visible absorption band with maxima at 512 and 519 nm was recorded after UV irradiation for the dissolved and neat ES-1, respectively (Figures 2 and S1), which resembles the behavior reported for pure DTE.^49–51^ It should be highlighted that ES-1_closed_, generated under these conditions, was not only composed of closed DTE units. Instead, a photostationary mixture was obtained, containing both open and closed DTE moieties, as also occurs for most diarylethenes.^52^ The composition of such mixtures typically depends on the excitation wavelength and the solvent. However, other experimental conditions, including concentration, are also important in our case, as the high amount of DTE units in neat ES-1 limits UV light penetration and, therefore, photocyclization efficiency in the absence of stirring. Interestingly, the formation of ES-1_closed_ can be reverted upon further irradiation with visible light to photoexcite the closed DTE units of the system (532 nm), which undergo light-induced ring-opening to recover the initial ES-1 state (Figures 2 and S1).

**Figure 2:**
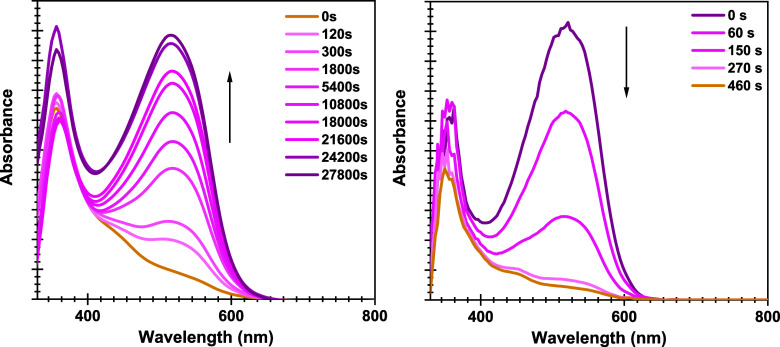
UV–vis absorption spectra of neat ES-1 until the PSS is reached after irradiation at: (left) 365 nm (27800 s) and (right) 532 nm (460 s). Measurements were conducted by depositing a thin layer of neat ES-1 (thickness ∼250 μm) on top of a transparent support.

According to the literature, DTE closed-to-open isomerization can also be triggered by electrochemical oxidation, which produces the corresponding radical cation of the closed isomer that spontaneously ring-opens to form the radical cation of the open state and eventually gets reduced to regenerate DTE_open_.^49–51^ These precedents prompted us to study the electrochromic behavior of ES-1 in solution and under neat conditions. To do so, the electrochemical properties of ES-1_open_ and ES-1_closed_ were investigated using cyclic voltammetry, enabling the determination of the potentials at which redox-induced ES-1_closed_ ring-opening should be conducted. Figure 3a shows the cyclic voltammogram of both states of ES-1, which were measured in solution with a supporting electrolyte (ACN/MeOH (5:1 v/v) + 0.1 M [TBA]­PF_6_). For ES-1_open_, a chemical irreversible two-electron oxidation wave^48^ was observed in the anodic region with a peak potential at *E*_pa_ = 1.33 V vs SCE. In agreement with the literature,^49,51^ an irreversible one-electron oxidation wave was registered at much lower potentials for ES-1_closed_ (*E*_pa_ = 0.66 V vs SCE), which can promote the ring-opening process of its DTE units (Figure 3b).

In light of these results, we explored the electroisomerization of ES-1_closed_ under oxidation potentials, which were chosen to be higher than that of *E*_pa_ of ES-1_closed_ (to trigger radical ion formation of its DTE_closed_ moieties) and lower than that of *E*_pa_ of ES-1_open_ (to prevent DTE_open_ degradation by oxidation). To monitor the redox-induced transformation of ES-1_closed_, spectroelectrochemical studies were performed, allowing the recording of the UV–vis absorption spectra during continuous oxidation. As shown in Figure 4, efficient color bleaching of ES-1_closed_ was observed upon oxidation in both solution (*E*_applied_ = 1.0 V vs SCE) and neat conditions (*E*_applied_ = 1.1 V vs SCE), a behavior that is consistent with redox-promoted ring opening of the closed DTE units in ES-1_closed_. This conclusion was corroborated by subsequent irradiation with UV light, which resulted in recoloration of these samples due to photocyclization of the open DTE moieties regenerated upon oxidation. It must be noted that redox-induced ES-1_closed_ back-isomerization took much longer under neat conditions (2800 s) than in solution (65 s), as expected due to the higher concentration of DTE units. Despite this, full recovery of the initial ES-1_open_ absorbance was not observed, even after such a long electrolytic time, suggesting that the rate of the redox-induced ring-opening process is significantly slow. Regarding the long-term reversibility of this ES in response to light or redox stimuli, no significant degradation of the sample was detected after several cycles of stimulation. The robustness of this photoelectrochromic diarylethene system can be attributed to the fully reversible and self-regenerating nature of its photochemical and electrochemical processes. Upon UV irradiation, the molecule switches from a colorless open form to a colored closed form, which reverts under visible light without structural degradation. The closed form can also be electrochemically oxidized to a radical cation, reopening spontaneously. Then, this open-ring radical cation can be reduced back to the neutral open form via an intermolecular electron transfer reaction with closed isomer molecules (Figure 3b). This redox cycle prevents the accumulation of degradation products and ensures that reactive intermediates are short-lived, resulting in a self-regenerating system suitable for long-term applications.

Although the photo- and electrochromic behavior observed for ES-1 was similar to that previously described for DTE,^49,50^ some significant differences were identified in terms of absorbance (closed-state λ_abs,max_ in the visible region) and electrochemical parameters (*E*_pa_), as summarized in Table 1. This table also includes the λ_abs,max_ and *E*_pa_ values previously reported for the three protonation states of pure DTE, namely, its diacid (DTE), monocarboxylate (DTE-COO^–^) and dicarboxylate (DTE-2COO^–^) forms, which slightly vary with the deprotonation degree of DTE.^49,50^

**Figure 3:**
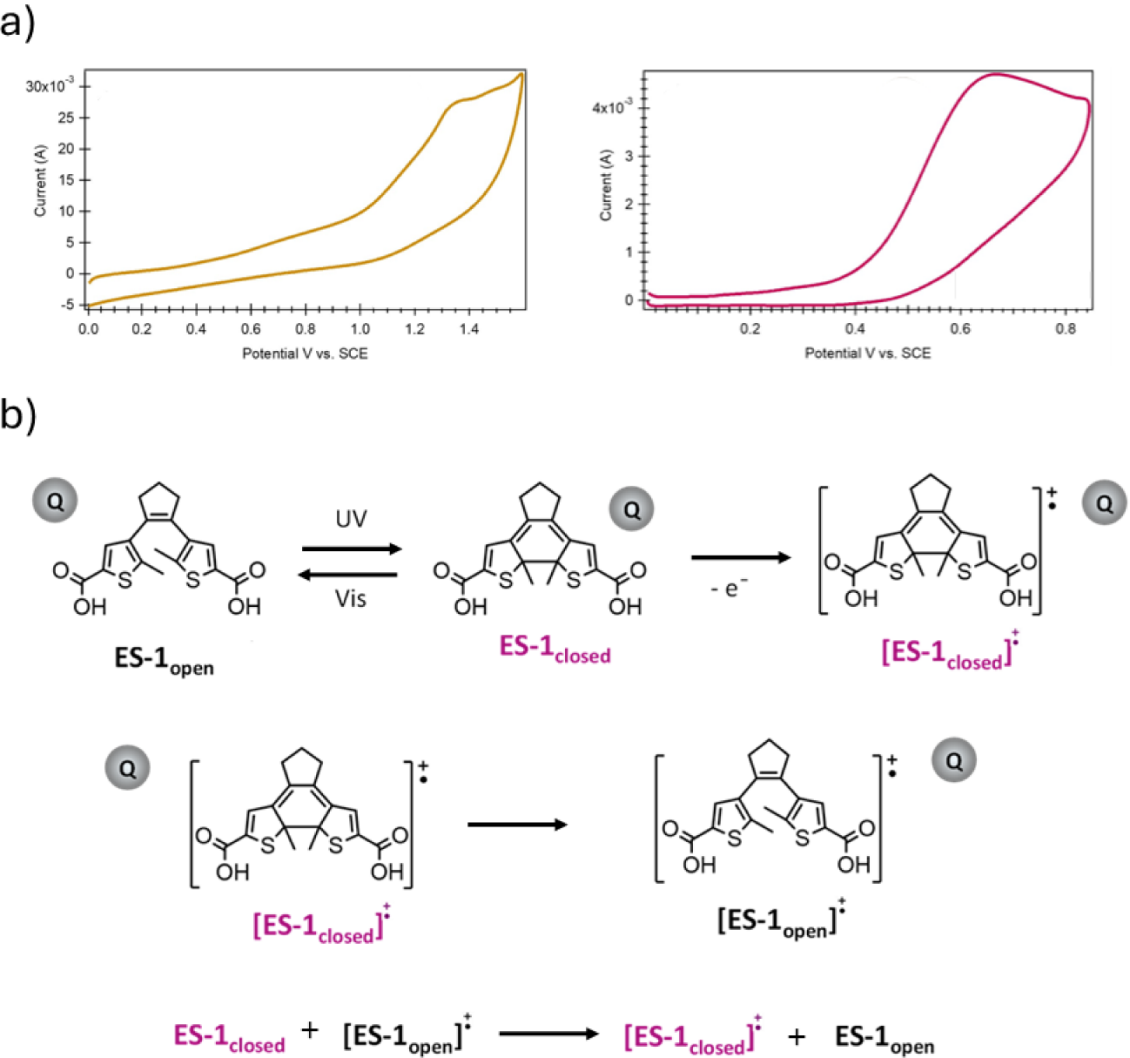
a) Cyclic voltammograms of a 3 mM solution of ES-1_open_ (left) and ES-1_closed_ (right) in ACN:MeOH (5:1 v/v) + 0.1 M [TBA]­PF_6_ at 0.2 V·s^–1^, comprising the 0/1.5/0 electrochemical window. The measurements were performed under N_2_ atmosphere in a 3-electrode configuration device, where platinum wire, platinum grid, and saturated calomel electrode were used as working, counter, and reference electrodes, respectively. ES-1_closed_ was prepared by previous irradiation of ES-1_open_ at 254 nm, which produced a PSS with around 31% of closed DTE units, according to the intensity of their oxidation wave at *E*_pa_ = 0.66 V vs SCE. b) Scheme of the photo- and electrochemical isomerization process for the DTE unit of ES-1, where Q represents the quadrol moiety.

**Figure 4:**
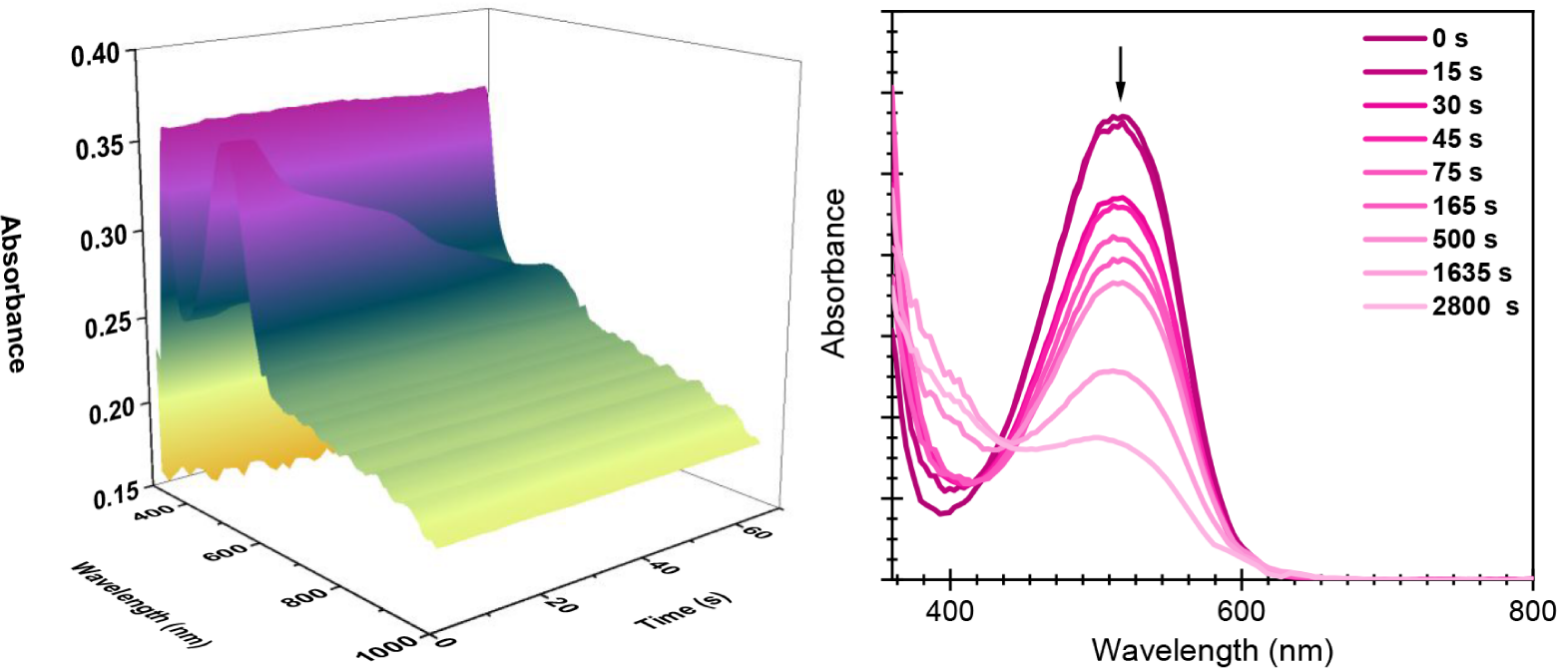
(Left) 3D graphical representation of the variation of the absorption spectrum of ES-1_closed_ (*c* = 3 mM) in ACN/MeOH (5:1 v/v) + 0.1 M [TBA]­PF_6_ solution when a constant potential of 1.0 V vs SCE was applied for 65 s. (Right) Variation of the UV–vis absorbance spectrum of neat ES-1_closed_ when a constant potential of 1.1 V vs SCE was applied for 2800 s.

**Table 1:** Photo- and Electrochemical Parameters for ES-1 and DTE^49,50^ in ACN Solutions

	ES-1		DTE		
Isomer	*E*_pa_ (V) vs SCE		Protonation state	*E*_pa_ (V) vs SCE^51^	
Open	1.33		DTE	1.44	
			DTE-COO^–^	1.12	
			DTE-2COO^–^	0.87	
					
	E_pa_ (V) vs SCE	λ_abs,max_ (nm)		*E*_pa_ (V) vs SCE^49^	λ_abs,max_ (nm)^50^
Closed	0.66	512	DTE	0.85	536
			DTE-COO^–^	0.57	533
			DTE-2COO^–^	0.33	498

The analysis of the *E*_pa_ values revealed a reduction in the peak oxidation potential of DTE in ES-1 relative to the pure diacid DTE, with a decrease of 8% for DTE_open_ and 22% for DTE_closed_. These findings indicate that the carboxylic groups of DTE interact significantly with the complementary moieties of Q in the closed state of ES-1, a behavior that is in agreement with the light-induced modulation of acidity previously reported for DTE.^50^ In particular, the acidity of pure DTE_closed_ was reported to be higher than that of DTE_open_, with 25- and 10-fold increments in the *K*_a_ acidity constant of their first and second ionization processes. Consequently, the hydrogen-bond donor capacity of the carboxylic acid groups of DTE should also increase upon photocyclization, thus accounting for the stronger interaction of DTE_closed_ with Q in ES-1. In other words, the introduction of the photoelectrochromic DTE unit into ES-1 allows modulation of the supramolecular interactions within the eutectic mixture, which might influence the thermal and electrical properties of the system.

### Thermal Analysis by Differential Scanning Calorimetry of the Eutectic System, ES-1

To investigate the effect of DTE isomerization on the thermal behavior of ES-1, DSC measurements were performed for neat Q, ES-1_open_ and ES-1_closed_. The latter was obtained through a previous photocyclization reaction of ES-1_open_, which leads to an equilibrium photostationary state (PSS) where both DTE_open_ and DTE_closed_ isomers coexist, as mentioned earlier. As a result, the differences that could be observed in the thermal properties of ES-1_open_ and ES-1_closed_ must be taken as a lower limit of the actual modulation amplitude between the fully open and closed forms of ES-1.

Figure 5 illustrates the thermograms obtained for ES-1_open_ and ES-1_closed_ in the second heating run after water removal, as well as for neat Q (first and second heating runs), which is the major component of the eutectic mixture. For the first heating cycle of neat Q (see inset of Figure 5), a glass transition process was observed at *T*_g‑onset_ = −33.4 °C, followed by an endothermal event associated with water evaporation (*T* = 20–120 °C). Removal of water caused the glass transition of Q to shift to higher temperatures (*T*_g‑onset_ = −28.8 °C) in the subsequent cooling and heating runs, which demonstrates the glass-forming ability of this compound and indicates that water acts as a plasticizer when it is hydrated. A similar thermal behavior was observed for the two states of ES-1: the addition of DTE to Q did not result in the appearance of other thermal events in the eutectic mixture, apart from a single glass transition process. Therefore, this suggests that both ES-1_open_ and ES-1_closed_ correspond to homogeneous mixtures of Q and DTE that also produce vitreous states upon cooling—a thermal response that was found to be independent of the heating and cooling rates tested (5, 10, and 20 °C min^–1^), with no visible degradation of the mixture up to 120 °C.

**Figure 5:**
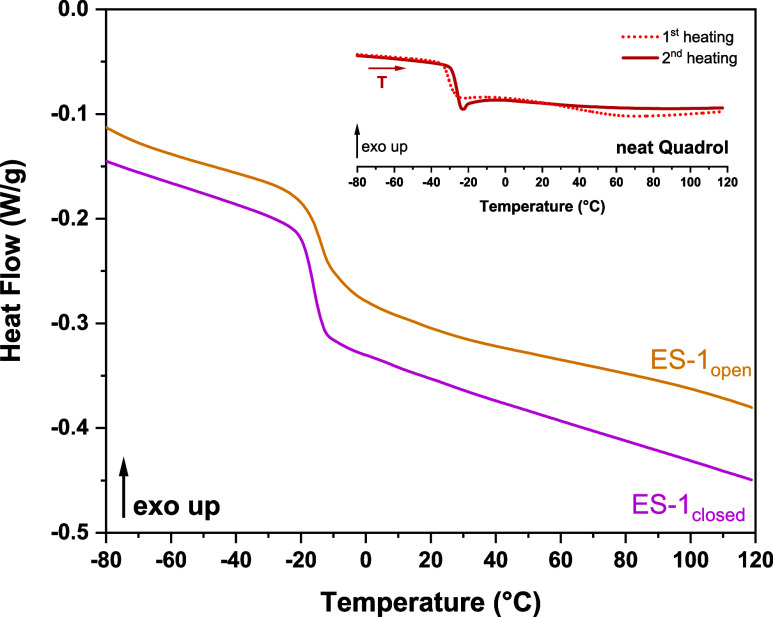
DSC curves obtained at 10 °C min^–1^ for dried ES-1_open_ and ES-1_closed_ (2nd heating run). Inset: thermograms of neat Q (1st and 2nd heating runs).

However, some significant differences can be detected between the DSC thermograms of Q and the two forms of ES-1, which are also illustrated by the thermal parameters given in Table 2: *T*_g‑onset_ values and the heat capacity change (Δ*C*_p_) measured for the first (hydrated samples) and second heating (dried samples) cycles of neat Q, ES-1_open_, and ES-1_closed_. On the one hand, we observed that the glass transition registered for dried Q presents an accentuated relaxation enthalpy, which is typically associated with the release of degrees of freedom upon heating that normally occurs after the sample has been in a nonequilibrium state below its glass transition temperature.^53^ In contrast, neither dried ES-1_open_ nor dried ES-1_closed_ exhibited such a relaxation enthalpy when *T*_g_ is exceeded under the same experimental conditions. This result suggests that the eutectic mixtures have a higher resistance to physical aging in their glassy state, which could originate from a more compact—i.e., less free volume—or rigid structure, probably due to Q–DTE interactions. The latter could also explain the differences measured for *T*_g‑onset_ between Q and the two isomers of ES-1. In particular, an increment of about 10 °C was registered for dried ES-1_open_ and ES-1_closed_ relative to neat Q, which may indicate that the additional hydrogen bonds between Q and DTE in these mixtures stabilize their glassy phase. As a result, more thermal energy would be required to activate their molecular motion through glass transition.

**Table 2:** Temperatures (*T*_g‑onset_) and Heat Capacity Variations (Δ*C*_p_) for the Glass Transition Detected on the First (Hydrated Samples) and Second Heating (Dried Samples) Runs of Neat Q, ES-1_open_, and ES-1_closed_ ^a^

	Hydrated (1^st^ heating run)		Dried (2^nd^ heating run)		
Compound	T_g‑onset_ (^o^C)	Δ*C*_p_ (J (g·^o^C)^−1^)	T_g‑onset_ (^o^C)	Δ*C*_p_ (J (g^o^C)^−1^)	Water content (%)^b^
Q	–33.4	0.73	–28.8	0.75	0.10
ES-1_open_	–30.4	0.66	–18.7	0.51	6.24
ES-1_closed_	–28.1	0.71	–19.6	0.73	2.51

a *T*_g‑onset_ was defined as the onset of heat flow change.

b The water content was estimated from the percentage of weight lost during the first calorimetric run.

Regarding the comparison between ES-1_open_ and ES-1_closed_, much smaller differences in *T*_g‑onset_ were determined (∼1 °C), which are within the error margin of our measurements. More significant differences were observed for the Δ*C*_p_ value associated with the glass transition of ES-1_open_ and ES-1_closed_: 0.51 J (g °C)^−1^ vs 0.73 J (g °C)^−1^, respectively. Such a variation suggests that ES-1_closed_ presents a more ordered glassy state as it gains more degrees of freedom with the glass transition. This observation could be correlated with two main factors: (i) the stronger hydrogen bonds expected between DTE_closed_ and Q according to the electro-optical measurements discussed above, and (ii) the structural changes associated with the isomerization of DTE, as DTE_closed_ exhibits a rigid conjugated planar structure that should enable additional π–π stacking interactions^54^ to take place in the glassy state of ES-1_closed_. In light of this, an even more compact and ordered molecular arrangement should be expected for ES-1_closed_ relative to ES-1_open_, which leads to a measurable variation in the thermal properties upon reversible photoisomerization between these two states.

### Electrical Properties by Dielectric Spectroscopy (DS) of the Eutectic System, ES-1

#### Electrical Conductivity

Similar to the thermal behavior, the effect of DTE isomerization on the conductivity profiles was also investigated through Dielectric Spectroscopy (DS) experiments for neat Q, ES-1_open_, and ES-1_closed_, assessing the frequency and temperature dependence of complex electrical conductivity σ*­(ω,*T*) and impedance Z*­(ω,*T*), in which both properties comprise a real and an imaginary component.^55,56^

In particular, the real part of complex electrical conductivity is related to charge migration, while the imaginary part corresponds to charge storage.^57,58^ In general, semiconducting disordered materials, such as DESs, exhibit a common profile for the real conductivity (σ′) plots, presenting different regimes that may or may not be frequency dependent.^55^ At lower temperatures and higher frequencies, σ′ depends on frequency, corresponding to a short-distance subdiffusive charge transport mechanism (alternating current conductivity) through back-and-forth motion over limited ranges.^26,59–61^ As temperature increases, a frequency-independent regime emerges as a plateau (direct current conductivity (σ_dc_) value), indicating the transition to a diffusive regime due to long-distance charge migration.^26,59–61^

For all of the samples, DS measurements were performed in both hydrated and dried states, the latter being obtained after a thermal treatment at 120 °C for 1 h. Figure 6 displays the conductivity spectra, collected from −100 to 120 °C, for dried ES-1_open_ and ES-1_closed_, while the data for their hydrated states and neat Q are plotted in Figures S2–S4.

**Figure 6:**
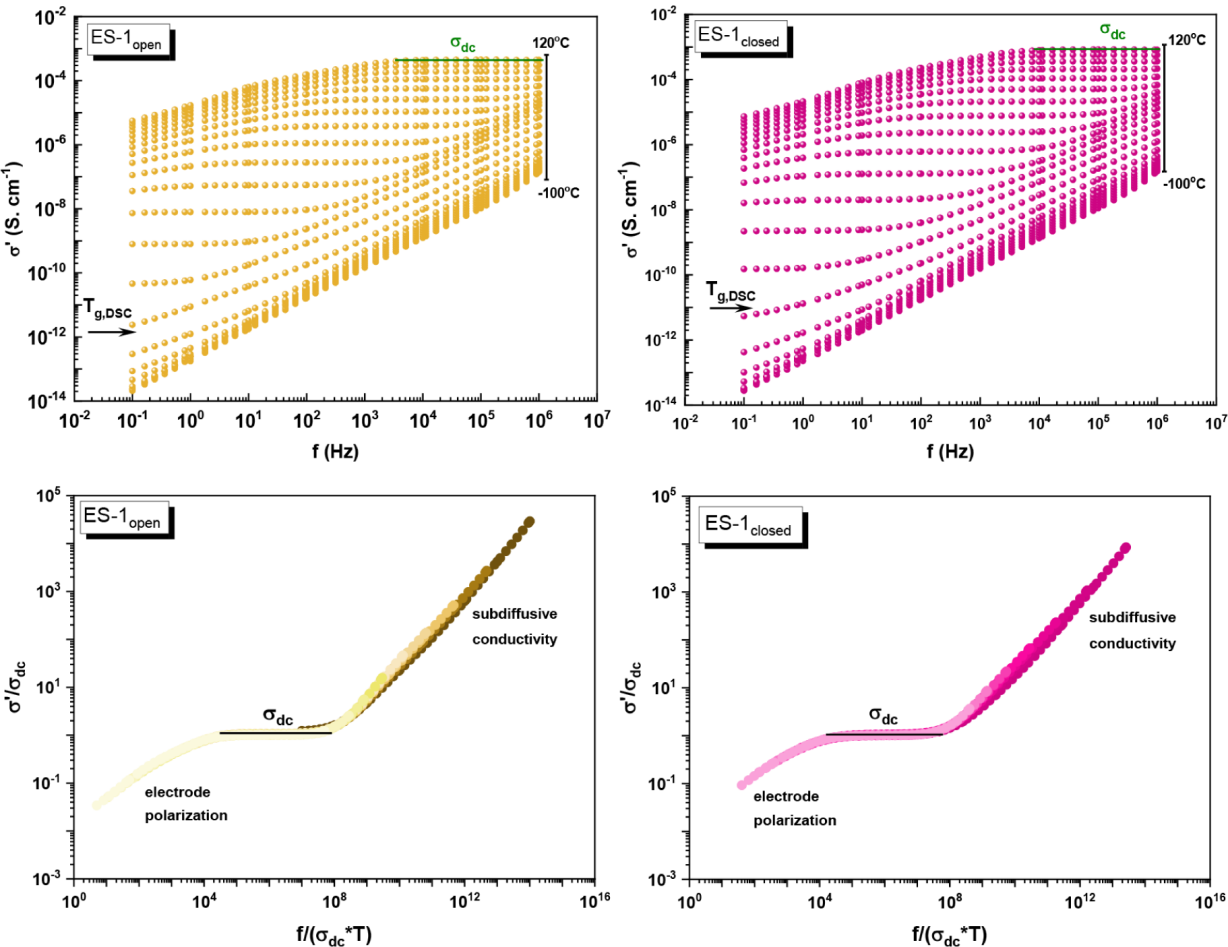
Real part of the complex conductivity (σ′) spectra and the master curves scaled, according to Summerfield equation, for dried ES-1_open_ and ES-1_closed_.

Similar to our previous works with DESs,^14,26^ the plateau in the σ′ plot of hydrated and dried ES-1_open_ as well as dried ES-1_closed_ was only observed at temperatures above the calorimetric glass transition (*T*_g,DSC_) (see arrow in Figure 6), indicating a relationship between the cooperative motions of the dynamical glass transition and the transport mechanism that governs the diffusive regime (σ_dc_).^59,62^ However, this behavior was not observed for hydrated ES-1_closed_. At higher temperatures and lower frequencies, for both samples in hydrated and dried states, a reduction of σ′ value is detected with the decrease in frequency, as charge carriers accumulate at the electrodes’ surface without discharging.^56,60^ This phenomenon is called electrode polarization. Furthermore, the obtained σ′ spectra for each material, acquired at different temperatures, can be superimposed according to the Summerfield master plot. This allows for better distinction between each above-mentioned regime in the respective conductivity profile (see Figure 6, bottom plots).^63^

Another relevant parameter to be considered in such materials is the fragility index (*m*), which describes the degree of curvature based on the steepness of the temperature dependence at *T*_g_ (Equation S3). For this parameter, higher *m* values indicate a greater variation in viscosity and conductivity with temperature at *T*_g_. Materials can be classified as strong (*m* ∼ 16) or fragile (*m* ≥ ∼200), depending on whether their transport properties resist more or less against temperature changes, respectively.^64^

Table 3 summarizes the σ_dc_ at *T*_g_ and at 25 °C, as well as the temperature range for which it was possible to use Jonscher’s equation, VFTH fit parameters, and the fragility index (*m*).

**Table 3:** σ_dc_ at *T*_g_, DSC and 25 °C, Applicable Temperature Range for Jonscher’s Equation, VFTH Fit Parameters, and Fragility Index

		Jonscher’s data			VFTH fit parameters			
	Compound	σ_dc_ (*T*_g,DSC_) [S cm^–1^]	σ_dc_ (25 ^o^C) [S cm^–1^]	[*T*_i_;*T*_e_]^a^ [^o^C]	σ_∞_[S cm^–1^]	*B* [K]	*T*_0_ [K]	*m* ^b^
Hydrated	Q	2.0 × 10^–14^	2.3 × 10^–8^	[−22; 100]	0.4	2163.1	169.3	38
	ES-1_open_	2.8 × 10^–12^	9.1 × 10^–6^	[−32; 38]	70.8	2070.5	168.2	51
	ES-1_closed_	3.9 × 10^–7^	3.3 × 10^–4^	[−60; 10]	37.2	1732.1	149.9	52
Dried	Q	1.0 × 10^–14^	2.2 × 10^–8^	[−16; 100]	0.5	2003.3	179.5	41
	ES-1_open_	6.0 × 10^–13^	1.4 × 10^–7^	[−14; 80]	55.0	2440.6	175.7	47
	ES-1_closed_	1.0 × 10^–11^	3.2 × 10^–7^	[−16; 52]	28.8	2176.5	179.8	49

a *T*_i_—Initial temperature and *T*_e_—end temperature represent the selected temperature range to carry out the experiment.

b Fragility index estimated at σ­(1/*T*) = 5 × 10^–13^ S cm^–1^.

Foreseeing future applications at room temperature, it can be useful to compare the results at *T* = 25 °C. In this context, higher conductivity values were observed for both hydrated and dried ES-1_open/closed_ when compared to neat Q. In the hydrated state, ES-1_closed_ exhibits higher conductivity than ES-1_open_, a difference that becomes more pronounced at *T*_g_. At this temperature, the σ_dc_ of ES-1_closed_ is 5 orders of magnitude higher when compared with the open-ring isomer, despite both isomers having comparable estimated water contents. Thus, water plays an important role, not so much associated with its content but with how the charge transport is assured. It is important to note that well below *T*_g_, when ES-1_closed_ is in the glassy state, long-distance charge migration still occurs, pointing to a water-assisted charge transport mechanism. In the case of the dried isomers, the σ_dc_ value of ES-1_closed_ (3.2 × 10^–7^ S cm^–1^) is two times higher than ES-1_open_ (1.4 × 10^–7^ S cm^–1^).

Considering that hydrated ES-1_closed_ exhibited anomalous conductivity behavior with respect to the location of its glass transition temperature, the fragility index (*m*, Equation S3) was instead determined from the steepness of σ­(1/*T*), at a fixed conductivity value of 5 × 10^–13^ S cm^–1^, to standardize the estimation criterion. This value represents the average conductivity measured at *T*_g_ for the other compounds, closely aligning with the *ad hoc* value identified by Leys et al. for a series of methylimidazolium ionic liquids.^65^ Furthermore, the *m* values obtained for both hydrated and dried samples allow us to classify these systems as strong glass formers.

#### Nyquist Plots

To gain a better understanding of the impact of DTE isomerization on relevant electrical properties for the development of electronic devices, additional studies were conducted by using impedance data obtained from dielectric measurements. Indeed, the frequency and temperature dependence of the complex electrical impedance (Z*­(ω,*T*)) provide information related to the electrical properties of the material and electrolyte/electrode interface.^66^ The so-called Nyquist plot, in which the symmetric of the imaginary part is plotted as a function of the real one, can exhibit two distinct regions: (i) at high frequencies, a semicircle, related to bulk ionic conduction, emerges, and (ii) at low frequencies, a tail is detected due to the electrode polarization effect.^67,68^

The Nyquist plots measured for dried ES-1_open_ and ES-1_closed_ at different temperatures are presented in Figure 7, while the data for neat Q are given in Figures S7 and S8. For temperatures above the glass transition, it is possible to observe a semicircle, whose diameter decreases with increasing temperature, indicating that the resistance to charge migration is reduced. With the increase in temperature, a tail associated with electrode polarization starts to emerge, which is more evident in this type of plot compared to isothermal spectra. More importantly, at the same temperature, dried ES-1_closed_ showed a lower semicircle diameter—i.e., lower resistance—than ES-1_open_, allowing better charge transport across the bulk electrolyte. Therefore, this result corroborates the conclusion that the electrical conductivity of ES-1 can be photo- and electromodulated upon DTE isomerization, thereby illustrating the ability to tune the properties of deep eutectic solvents through the application of external stimuli.

**Figure 7:**
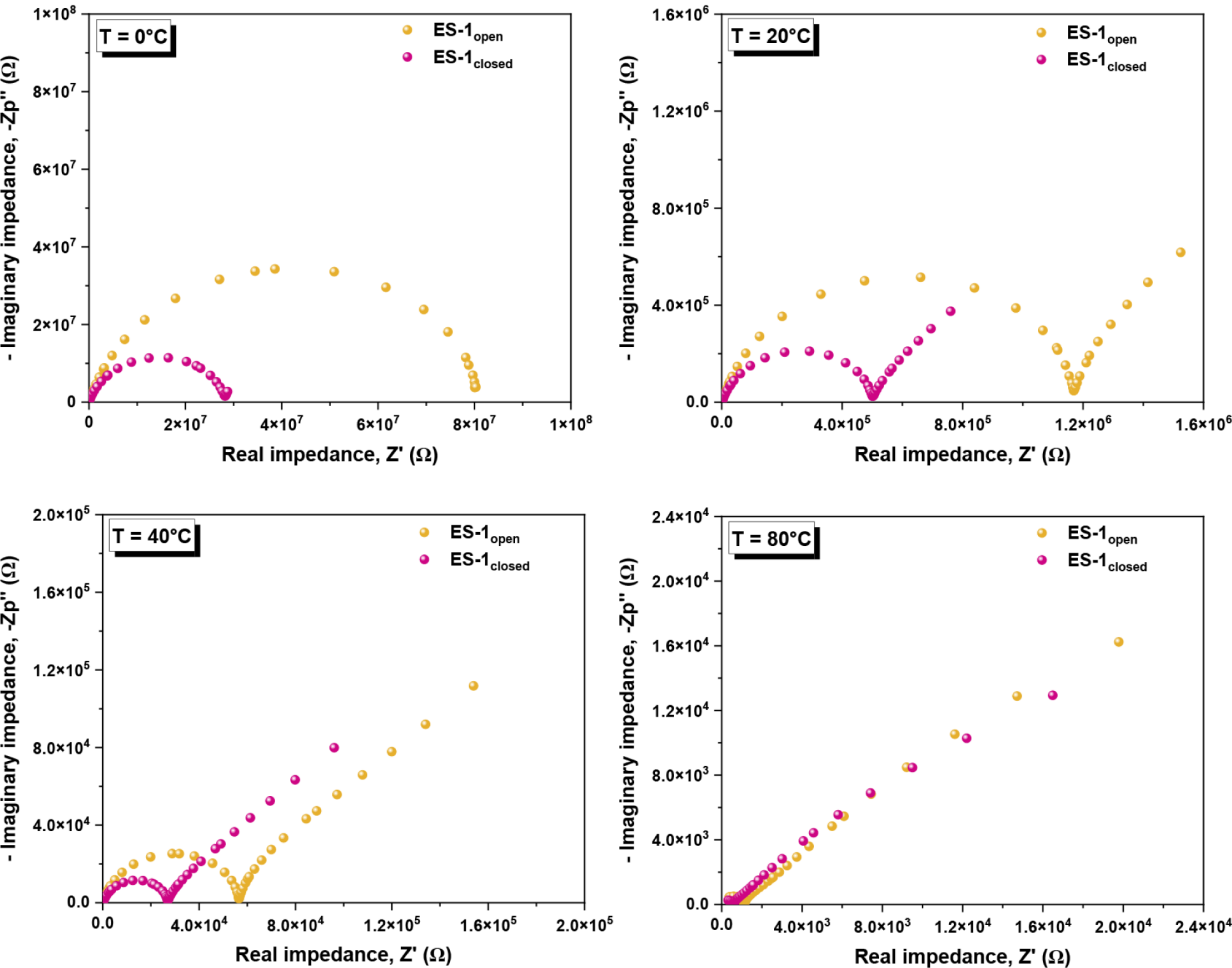
Nyquist plots for dried ES-1_open_ and ES-1_closed_ at different temperatures: 0 °C, 20 °C, 40 °C, and 80 °C.

## Conclusions

Smart responsive systems are attracting special interest due to their capacity to abruptly change their properties in response to external stimuli, such as light and electrical currents. Herein, we aim to prepare a new responsive system based on deep eutectic solvents, comprising a photo- and electrochromic dicarboxylic diarylethene derivative and quadrol. The prepared system reveals a variation in coloration from yellowish to pink-red under UV irradiation due to the photoisomerization of DTE, which can be reverted with visible light or oxidative electrolysis. Interestingly, this process also alters the strength of the hydrogen bond between DTE and Q, resulting in an externally controlled change in the thermal and electrical properties of the eutectic mixture. Indeed, calorimetric analyses showed that ES-1_open_ and ES-1_closed_ are good glass formers, suggesting that the hydrogen bonds between Q and DTE in these mixtures stabilize their glassy phase, vitrifying at higher temperatures compared to neat Q. Notably, a significant difference in heat capacity was observed between the two isomers of ES-1, indicating that ES-1_closed_ exhibits a more ordered glassy state, as it gains more degrees of freedom with the transition from the glassy to the supercooled liquid phase. Moreover, according to dielectric data, ES-1_closed_ also showed higher electrical conductivities than ES-1_open_, both at cryogenic temperatures slightly above the glass transition and at room temperature. These results were corroborated by Nyquist plots, where ES-1_closed_ presented a lower resistance, suggesting that it allows better charge transport across the bulk electrolyte than ES-1_open_. Overall, this work demonstrates the potential of developing stimuli-responsive eutectic mixtures, as the external control of their physicochemical properties—e.g., color, conductivity—can find applications in a wide range of fields, such as smart solvent systems for extraction and separation and switchable catalysis. In this sense, the ability of these responsive deep eutectic solvents (RDESs) to reversibly alter color, polarity, and hydrogen bonding strength under photochemical or electrochemical stimuli could be used not only for the selective and tunable extraction of target compounds but also to modulate catalytic activity or selectivity on demand, paving the way for more efficient and controllable chemical processes.

## Supplementary Material



## Notes

Experimental techniques used to characterize the eutectic system. These comprise photo- and electro-induced response measurements, electrical properties by dielectric spectroscopy, and impedance characterization through Nyquist plots (PDF)
