# Supplementary material for: A Switchable Deep Eutectic System Based on Diarylethene
Source: ACS Omega. 2025 Oct 21;10(43):50951–61. doi: 10.1021/acsomega.5c04216 (PMC12593998; doi:10.1021/acsomega.5c04216)
Supplement: Supplementary file 1 [file ao5c04216_si_001.pdf]

Supporting Information:

A switchable deep eutectic solvent based on diarylethene

Hugo Cruz,[a,b,*] Noémi Jordão,[c] Sara Santiago,[c] Silvia Mena,[c] Andreia F. M. Santos,[a] M. Teresa Viciosa,[d] Karolina Zalewska,[a] Luis C. Branco,[a] Jordi Hernando,[c,*] and Gonzalo Guirado[c,*]

[a] LAQV-REQUIMTE, Department of Chemistry, NOVA School of Science and Technology, NOVA University of Lisbon, 2829-516 Caparica, Portugal
[b] INL – International Iberian Nanotechnology Laboratory, Av. Mestre José Veiga, 4715-330 Braga, Portugal
[c] Departament de Química, Universitat Autònoma de Barcelona, Cerdanyola del Vallès, Barcelona, 08193, Spain
[d] Centro de Química Estrutural, Institute of Molecular Sciences, Instituto Superior Técnico, University of Lisbon, Av. Rovisco Pais, 1049-001 Lisbon, Portugal

e-mail: hugogdsc@gmail.com; jordi.hernando@uab.cat; gonzalo.guirado@uab.cat

Table of Contents:

1. Photo- and electroinduced responses of the Eutectic System, ES-1 2
2. Electrical Properties by Dielectric Spectroscopy (DS) 2
   2.1. Real part of the complex conductivity (σ') spectra 2
   2.2. Master curves scaled, according to Summerfield equation 3
   2.3. Determination of σdc: 3
   2.4. Activation Map with the estimated σdc from Jonscher's fits 4
   2.5. Determination of fragility index (m): 4
3. Nyquist Plots 5

1. Photo- and electroinduced responses of the Eutectic System, ES-1

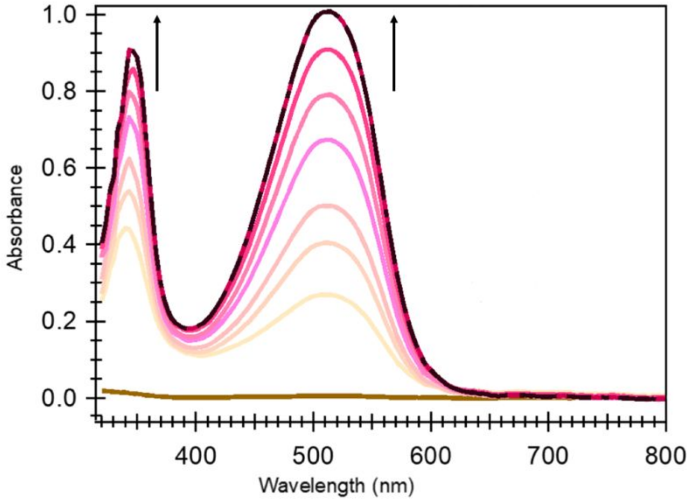

Figure S1. UV-Vis absorption spectral variations upon irradiation of 3mM solution of ES-1_{open} at 256 nm (300 s) until the photostationary state (PSS) is achieved.

2. Electrical Properties by Dielectric Spectroscopy (DS)

2.1. Real part of the complex conductivity (σ') spectra

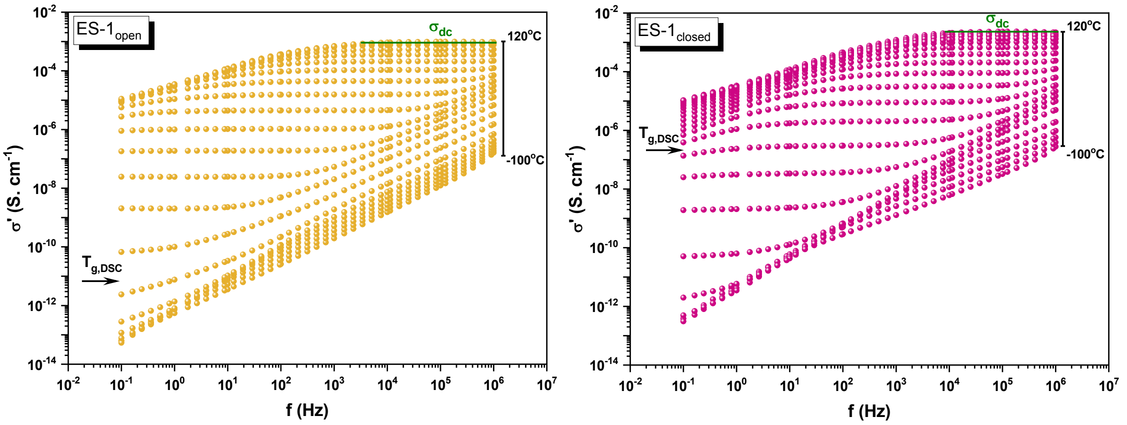

Figure S2. Real part of the complex conductivity (σ_{dc}) spectra for hydrated ES-1_{open} and ES-1_{closed}.

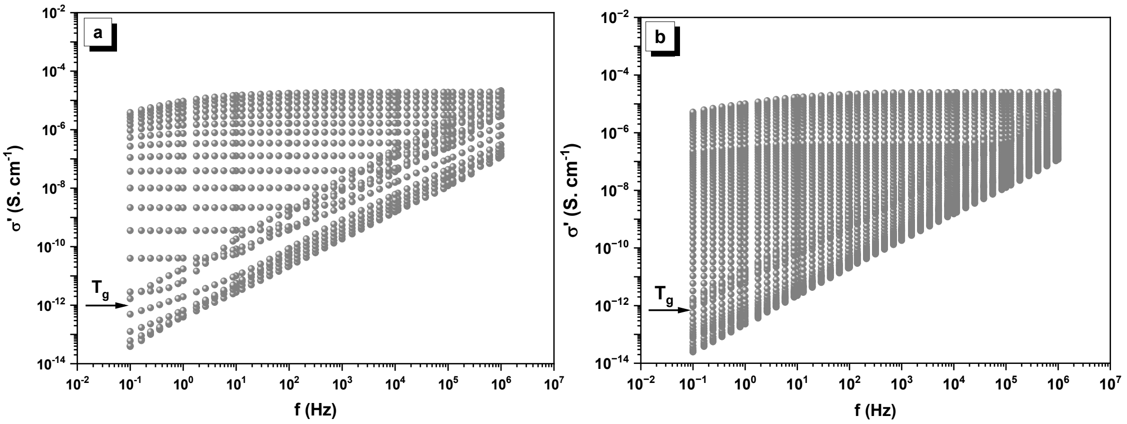

Figure S3. Real part of the complex conductivity (σ_{dc}) spectra for (a) hydrated and (b) dried quadrol (Q).

2.2. Master curves scaled, according to Summerfield equation

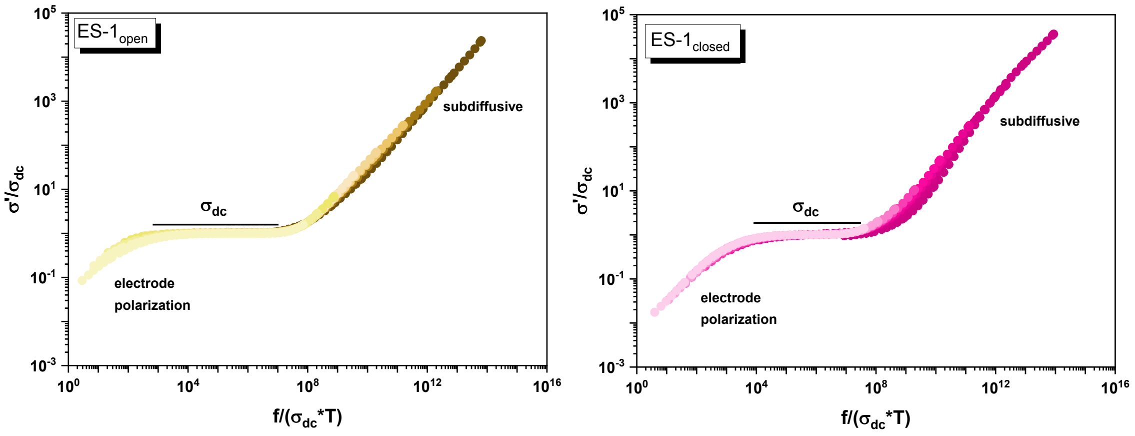

Figure S4. Master curves scaled, according to Summerfield equation, for hydrated ES-1_{open} and ES-1_{closed}.

2.3. Determination of σ_{dc}:

Aiming to estimate the value of σ_{dc}, all isotherms have been fitted by the Jonscher's equation, which is defined in Equation S1 (see Figure S5).

$$\sigma'(\omega) = \sigma_{dc}\left[1 + \left(\frac{\omega}{\omega_c}\right)^s\right] \qquad \text{Equation S1}$$

where s ($0.5 \leq s \leq 1$) is a material and temperature dependent parameter and ω_{cross} is the angular frequency, where the plateau bends off, separating diffusive from sub-diffusive regimes.

The estimated $\sigma_{dc}(T)$ values from Jonscher's fitting were plotted in function of temperature reciprocal (see Figure 7), showing a non-Arrhenian curved temperature dependency, which can be easily described by the Vogel-Fulcher-Tammann-Hesse (VFTH) function (Equation S2).

$$\sigma_{dc}(T) = \sigma_\infty exp\left(-\frac{B}{T-T_0}\right) \qquad \text{Equation S2}$$

Where σ_∞ is the high temperature limit of the σ_{dc} conductivity, B is the curvature, and T_0 is the Vogel temperature.

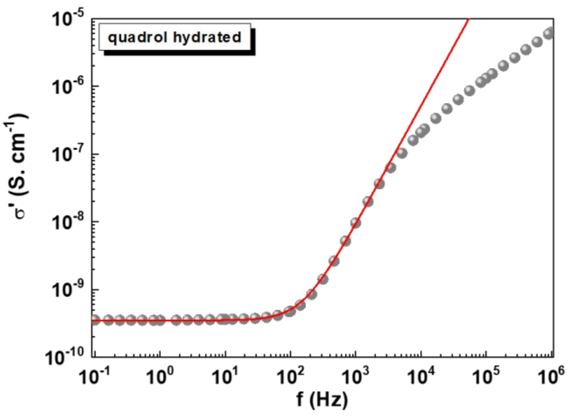

Figure S5. Example of an isothermal that has been fitted by the Jonscher's equation for hydrated quadrol.

2.4. Activation Map with the estimated σ_{dc} from Jonscher's fits

Figure S6 comprises the activation map with the estimated σ_{dc} from Jonscher's fits in function of temperature reciprocal. The obtained data was further simulated by VFTH, which is also included in Figure S5 as solid lines.

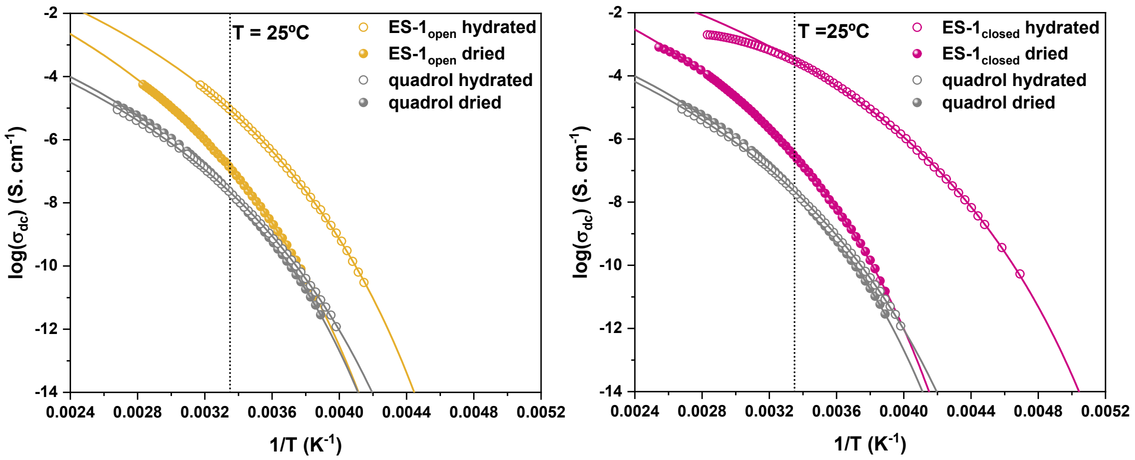

Figure S6. Temperature dependence of σ_{dc} obtained from the fits with Jonscher's equation for: (left) ES-1_{open} and Q; (right) ES-1_{closed} and Q. For all samples, hydrated and dried data are included. The $\sigma'(f)$ solid lines correspond to the VFTH fit of the experimental data. the vertical dotted lines indicate T = 25°C.

2.5. Determination of fragility index (m):

The fragility index (m), which describes the curvature degree by the deepness of the temperature dependence at T_g, can be determined as indicated in equation S3.

$$m = \left(\frac{d(\log(\sigma))}{d\left(\frac{T_g}{T}\right)}\right)_{T=T_g} = \frac{BT_g}{ln10(T_g - T_0)^2} \qquad \text{Equation S3}$$

3. Nyquist Plots

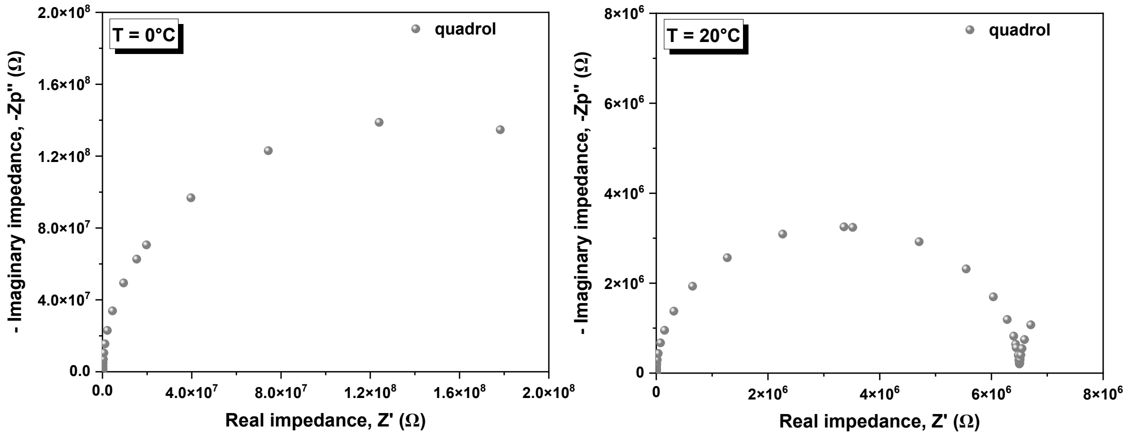

Figure S7. Nyquist plot for dried quadrol (Q) at different temperatures: 0 °C and 20 °C.

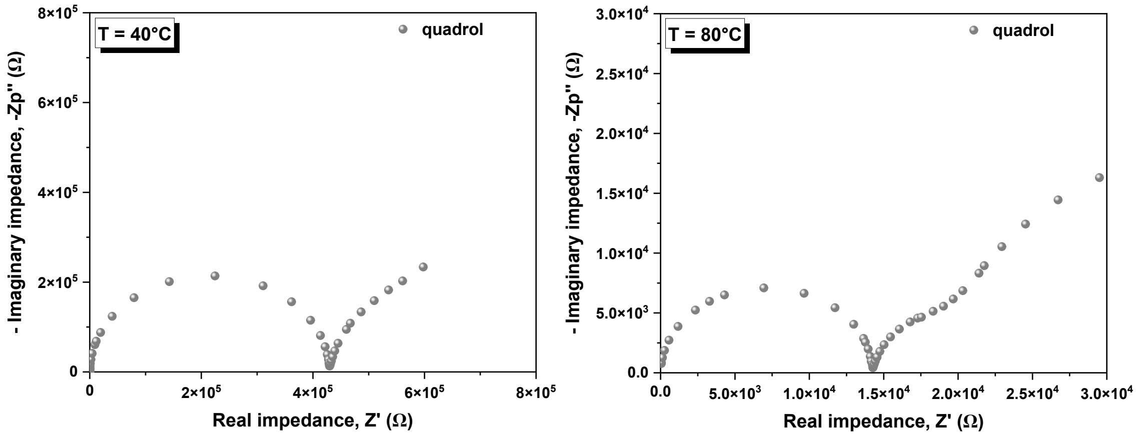

Figure S8. Nyquist plot for dried quadrol (Q) at different temperatures: 40 °C and 80 °C.
